# Genome-Wide Analysis of the Chromatin Composition of Histone H2A and H3 Variants in Mouse Embryonic Stem Cells

**DOI:** 10.1371/journal.pone.0092689

**Published:** 2014-03-21

**Authors:** Masashi Yukawa, Tomohiko Akiyama, Vedran Franke, Nathan Mise, Takayuki Isagawa, Yutaka Suzuki, Masataka G. Suzuki, Kristian Vlahovicek, Kuniya Abe, Hiroyuki Aburatani, Fugaku Aoki

**Affiliations:** 1 Department of Integrated Biosciences, Graduate school of Frontier Sciences, The University of Tokyo, Kashiwa, Japan; 2 Bioinformatics Group, Division of Biology, Faculty of Science, Zagreb University, Zagreb, Croatia; 3 Mammalian Cellular Dynamics Team, Bioresource Center, RIKEN Tsukuba Institute, Tsukuba, Japan; 4 Genome Science Division, Research Center for Advanced Science and Technology, The University of Tokyo, Tokyo, Japan; 5 Department of Medical Genome Science, Graduate school of Frontier Sciences, The University of Tokyo, Kashiwa, Japan; Florida State University, United States of America

## Abstract

Genome-wide distribution of the majority of H2A and H3 variants (H2A, H2AX, H2AZ, macroH2A, H3.1, H3.2 and H3.3) was simultaneously investigated in mouse embryonic stem cells by chromatin immunoprecipitation sequencing. Around the transcription start site, histone variant distribution differed between genes possessing promoters of high and low CpG density, regardless of their expression levels. In the intergenic regions, regulatory elements were enriched in H2A.Z and H3.3, whereas repeat elements were abundant in H2A and macroH2A, and H3.1, respectively. Analysis of H2A and H3 variant combinations composing nucleosomes revealed that the H2A.Z and H3.3 combinations were present at a higher frequency throughout the genome than the other combinations, suggesting that H2A.Z and H3.3 associate preferentially with each other to comprise the nucleosomes independently of genome region. Finally, we found that chromatin was unstable only in regions where it was enriched in both H2A.Z and H3.3, but strongly quantified stable in regions in which only H3.3 was abundant. Therefore, histone variant composition is an important determinant of chromatin structure, which is associated with specific genomic functions.

## Introduction

The eukaryotic genome consists of genes and intergenic regions, which include enhancers, insulators, repeat elements, *etc*. These genomic components play roles in various genomic functions, such as gene expression, DNA replication and maintenance of genome stability. Emerging evidence from epigenetic studies revealed that various genome regions contain specific modifications on their nuclear core histones, which are involved in functional regulation [1], [2]. Furthermore, recent studies showed that histone variants also play important roles in the regulation of genomic function [3]–[8]. Nuclear core histones H2A and H3 exist as several variants: H2A has the canonical H2A, H2A.X, H2A.Z, macroH2A (mH2A) and H2A.Bbd; H3 has H3.1, H3.2, H3.3, CENP-A and H3t [9]. These variants have been suggested to have specific functions in the regulation of gene expression and genome stability.

Of the H2A variants, canonical H2A is the most abundant in chromatin [10]. Although its function is unclear, recent studies suggest that canonical H2A is involved in gene silencing [11]. H2A.X is a highly conserved H2A variant. It is involved in the DNA damage repair pathway [12], [13]. H2A.X is present at higher levels in mouse embryonic stem (mES) cells than in neuronal precursor cells, which are differentiated from mES cells [14]. H2A.X is preferentially incorporated into the nucleus of totipotent one-cell stage embryos [15]. These results suggest that H2A.X is involved in regulation of the undifferentiated state and cell proliferation. H2A.Z is a highly conserved variant, as is H2A.X. It is enriched in promoters and is associated with transcriptional regulation [16]. H2A.Z enrichment in promoters is negatively correlated with CpG methylation in plant and mammalian cells [17], [18]. mH2A is a vertebrate-specific histone H2A variant and contains a large C-terminal domain, which is termed a macro domain [9], [19], [20]. mH2A is implicated in transcriptional repression during X chromosome inactivation [21]. Recent studies have shown that mH2A enrichment in gene bodies is negatively correlated with gene expression [6], [22].

Of the H3 variants, H3.3 is the evolutionarily conserved replacement H3 [23]. H3.3 seems to be involved in the activation of gene expression. It is modified by active marks (*i.e.*, H3K4me3, H3K36me3) and acetylation of various N-terminal lysines [24], [25], and is enriched in the promoters and gene bodies of actively expressed genes [7]. It is also enriched in enhancers [7], [26]. H3.1 and H3.2 have similar amino acid sequences; they differ in only a single residue [27]. Therefore, no antibody that can distinguish them is available, which has hampered both the clarification of their distribution in the genome and their function. However, mass spectrometry analysis revealed differences in their N-terminal modifications. H3.1 is preferentially acetylated and dimethylated on lysines 14 and 9, respectively, while H3.2 is preferentially subjected to trimethylation on lysine 27 [24], [25]. Recently, genome-wide analyses of H3.2 in *Arabidopsis thaliana* showed that H3.2 enrichment in genes is negatively correlated with their expression levels, and H3.2 was deposited abundantly in regions with methylated CpG and H3K9me2, as well as H3K27me3 [28], [29]. These results suggest that H3.2 is involved in the formation of heterochromatin structure. However, because *Arabidopsis thaliana* does not possess H3.1, the difference in the function of H3.1 and H3.2 remains to be elucidated.

Although several studies have assessed the genome-wide distributions of histone H2A and H3 variants, most examined only a single variant [6], [22], [30], [31]. Because these studies used different species and cell types and were conducted using different protocols, it is difficult to integrate their data to gain a comprehensive understanding of the H2A and H3 variant compositions of various genome regions. Furthermore, few studies have investigated the combination of H2A and H3 variants. One report showed that nucleosomes containing H3.3 and H2A.Z were less stable than those containing H3.3 and H2A [32]. Therefore, the function of a nucleosome could vary according to changes in its H2A and H3 variant composition. Thus, conducting a genome-wide analysis of H2A and H3 variant combinations will provide an understanding of the mechanism underlying the regulation of various genomic functions. Although only a single report has shown that nucleosomes containing H2A.Z and H3.3 are enriched at promoters, enhancers, and insulators [26], other combinations of H2A and H3 variants have not been investigated.

In the present study, we examined the distribution of the majority of genomic H2A and H3 variants in mES cells using chromatin immunoprecipitation (ChIP) followed by high-throughput sequencing. We found that the enrichment of histone variants in gene regions (promoter and gene body) differed depending on gene expression level. Further, the enrichment was markedly different around the transcription start sites (TSSs) of genes with high- and low-CpG promoters. Furthermore, specific H2A and H3 variant compositions were observed in various regulatory and repeat elements. Also, H2A.Z and H3.3 associated preferentially with each other throughout the genome. Taken together, our results suggest that specific H2A and H3 variant compositions regulate the functions of various genome regions.

## Materials and Methods

### ZHBTc4 mouse ES cell culture

Feeder-free ZHBTc4 ES cells [33] were maintained on gelatin-coated dishes with ES medium, which consisted of Glasgow Minimal Essential Medium (GMEM; Sigma-Aldrich, G5154) supplemented with 2 mM GlutaMAX I (Invitrogen, cat#35050-061), 110 μM 2-mercaptoethanol (Invitrogen, cat#21985-023), 1× MEM non-essential amino acids solution (NEAA; Invitrogen, cat#11140-050), 25 U/mL of penicillin-streptomycin (Invitrogen, cat#15140-122), 11 μM sodium pyruvate (Invitrogen, cat#11360-070), 14% (v/v) KnockOut Serum Replacement (KSR; Invitrogen, cat#10828-028), 1% (v/v) fetal bovine serum (FBS; Invitrogen, cat#16141-079), and 1,000 U/mL leukemia inhibitory factor (LIF; Millipore, cat#ESG1107). Blasticidin (1 mg/mL; Funakoshi, cat#KK-400) was added to the medium to maintain the pluripotency of the cells. To induce differentiation of the cells, the medium was replaced with ES medium without blasticidin, supplemented with tetracycline (Tc; Sigma, cat#T7660), and cultured for 72 h [33].

### Plasmid construction and production of transgenic cell lines with Flag-tagged histone variants

A pCAGGS vector was used to generate transgenic ES cell lines constitutively expressing Flag-histone variants (H2A, H2A.X, H2A.Z, mH2A, H3.1, H3.2, and H3.3). A DNA fragment encoding a histone variant tagged with Flag at its N-terminal region was inserted into the EcoRI site of the pCAGGS vector. The puromycin N-acetyl-transferase (*Pac*) gene obtained by SalI digestion of pCre-Pac was inserted into the SalI site of the vector. The vector coding a Flag-histone variant was transfected into ZHBTc4 cells by lipofection, and cells were selected in ES medium using 100 μg/mL of puromycin. A single colony of cells for each variant was picked up, cultured and subjected to ChIP-sequencing analysis. The difference in the genome distribution of a Flag-histone variant among the cell lines was assessed using the Flag-H2A.X cell lines. ChIP-quantitative polymerase chain reaction (qPCR) analysis of several genes showed no significant difference among the four cell lines examined (Figure S1).

Flag-tagged protein expression did not seem to affect ES cell properties, as no difference in the morphology of wild-type cells and those expressing Flag-tagged variants was observed (Figure S2). Furthermore, cells expressing Flag-tagged protein differentiated into trophectoderm like cells as well as the wild-type ones. Cluster analysis using gene expression profiles showed that the gene expression patterns of Flag-tagged-protein-expressing cells were not significantly altered from that of the wild-type cells. All types of undifferentiated cells expressing Flag-H2A and H3 variants were classified into the same cluster as the undifferentiated wild-type cells, while all differentiated cells clustered separately, regardless of the time after induction of differentiation (Figure S3).

### RNA isolation and RNA sequencing

Following the TruSeq RNA Sample Preparation Guide, total RNA was isolated from ZHBTc4 ES cells without polyA selection at 0 and 72 h after the induction of differentiation. RNA was fragmented by heat treatment and then subjected to first and second strand cDNA synthesis, amplified, and used for library construction. The library thus prepared was applied to a Genome Analyzer IIx (Illumina), following the manufacturer's protocols, and high-throughput sequencing was carried out. Sequences (35 bp) were aligned against the cDNA sets from Ensemble GRC38 using Bowtie. The number of mapped reads per kilobase of exon per million reads (RPKM) for each gene was calculated using the following formula: RPKM  =  (gene read number/(total read number × gene length)) ×10^9^.

### ChIP and sample preparation for sequencing

ES cells were cultured in 10-cm plates to ∼80% confluence (1.2×10^7^ cells per plate). One plate was used per ChIP aliquot. Formaldehyde solution (50 mM HEPES-KOH [pH 7.5], 100 mM NaCl, 1 mM EDTA [pH 8.0], 0.5 mM EGTA [pH 8.0], 11% formaldehyde) was directly added to culture media at a final concentration of 1% for fixation. After 10-min incubation at room temperature, a glycine solution was added to the plates at a final concentration of 125 mM, and the plates were incubated at room temperature for 10 min to stop fixation. Cells were transferred to 15 mL tubes and washed twice with cold phosphate buffered saline (PBS). All buffers used in the following procedures were supplemented with protease inhibitor solution (final concentration 1×; complete protease inhibitor cocktail tablet; Roche) before use. Cells were suspended in lithium borate (LB) buffer (10 mM Tris-HCl [pH 8.0], 100 mM NaCl, 1 mM EDTA [pH 8.0], 0.5 mM EGTA [pH 8.0], 0.1% Na-deoxycholate, 0.5% N-lauroylsarcosine), and sonicated (30 s pulse +1 min break) seven times on ice using a SONIFER 250 (Branson) to obtain 150–300-bp chromatin fragments. Triton X-100 was added into the tubes to a final concentration of 1%, and then the tubes were centrifuged (15,000 rpm, 4°C, 10 min) to obtain the supernatant used for immunoprecipitation. For the input sequencing, DNA was purified—without being immunoprecipitated—from cells expressing Flag-mH2A. To bind the antibodies to beads, 50 μL of Dynabeads Protein G (Invitrogen) were washed twice with PBS, 5 μg of the antibody in blocking solution (0.5% bovine serum albumin [BSA]/PBS) was added and gently flipped overnight at 4°C. Anti-FLAG M2 (Sigma, cat# F1804), anti-H3K4me3 (Millipore, cat#07-473), anti-H3K27me3 (Millipore, cat#07-449), and anti-H3K36me3 (Abcam, cat# ab9050) antibodies were used for immunoprecipitation. After three PBS washes, the antibody-bound beads were added to the supernatant containing the chromatin fragments, and gently flipped overnight at 4°C. The beads were washed once with a low salt buffer (20 mM Tris-HCl [pH 8.0], 150 mM NaCl, 2 mM EDTA [pH 8.0], 0.1% SDS, 1% Triton X-100), twice with a high salt buffer (20 mM Tris-HCl [pH 8.0], 400 mM NaCl, 2 mM EDTA [pH 8.0], 0.1% SDS, 1% Triton x-100), five times with RIPA buffer (50 mM HEPES-KOH [pH 7.5], 500 mM LiCl, 1 mM EDTA [pH 8.0], 1% NP-40, 0.7% Na-Deoxycholate), and once with TE (pH 8.0) supplemented with 50 mM NaCl. To elute the chromatin, the beads were suspended in elution buffer (50 mM Tris-HCl [pH 8.0], 10 mM EDTA [pH 8.0], 1% SDS), and incubated at 65°C for 15 min. After centrifugation (15,000 rpm, room temperature, 15 min), the supernatant was collected and incubated overnight at 65°C to de-crosslink the chromatin. At this time, the samples for input sequencing were added to the elution buffer and then subjected to the same treatments as those for the immunoprecipitated samples in the following procedures. After de-crosslinking, RNase A (25 mg, Roche) was added to all samples, which were incubated at 37°C for 2 h, followed by the addition of proteinase K (20 mg/mL; Takara Bio) and incubation at 55°C for 2 h. Finally, DNA was isolated by the phenol-chloroform method followed by ethanol precipitation; isolated DNA was dissolved in Milli-Q water. Adapters were ligated to both ends of the DNA following the Illumina protocol for “Preparing Samples for ChIP Sequencing of DNA.” The ligated DNA was subjected to electrophoresis, and 250–400-bp DNA fragments were excised from the gel and purified. The purified DNA was subjected to PCR amplification following the Illumina protocol and then sequenced using the Illumina Genome Analyzer (Illumina).

### Sequencing and read mapping

DNA fragments obtained from ChIP were applied to the deep sequencer following the manufacturer's protocol. Sequences (36 bp) obtained by high-throughput sequencing were aligned against the mouse reference genome (mm9) using Bowtie. The total number of uniquely mapped reads in each sample is shown in Table S2.

### Determination of the enrichment of each histone variant at a single-nucleotide resolution

Uniquely mapped 36-bp sequences in each ChIP sample were extended to 150–300 bp, which is the length appropriate for sequencing without adapters. At each mapped nucleotide position, the number of reads mapped there ×1,000,000/total number of reads was calculated. For input samples, the calculated value at each position was added to the minimum calculated value, except for zero, to avoid a value of zero at any position. The calculated values of ChIP samples were divided by those of input samples to determine the enrichment at each nucleotide position in the genome.

### Preferential combinations of H2A and H3 variants

The whole genome was compartmentalized into 150-bp bins (Figure S4). The ratio of the number of reads in a bin to the averaged read number of 3,333 bins (total length, ∼500 kb) around the corresponding bin was calculated for each variant, and the variant with the highest ratio value was selected. The read number of the selected variant was analyzed statistically using a Poisson distribution with the value of λ, which was determined by multiplying the averaged read number of the selected variant by the second highest ratio in the corresponding bin. If this was significant (FDR<0.05), the bin was judged to be enriched for that variant. The nucleosome around a bin enriched for specific H2A and H3 variants was considered to be preferentially composed of these two variants.

### Determination of major isoform among transcripts from a gene

To determine major isoform among transcripts from a gene, we deterimined the transcript with the highest RPKM value in transcripts from a gene registered in Ensemble (GRC38) as a major isoform of the gene.

### Gene classification according to CpG density in the promoter

Genes registered in Ensemble (GRC38) were classified into HCP, ICP, and LCP groups as described by Mikkelsen *et al.* [34].

### CpG islands

The CpG island annotation file was downloaded from the UCSC Genome Bioinformatics site (http://genome.ucsc.edu/), and used for the determination of CpG islands.

### Definition of TFBS

ChIPseq of transcription factor (TF) and CTCF data were obtained from Chen *et al*. [35]. TF and CTCF binding sites were identified using Cisgenome peak caller with cutoff (FDR<0.01). The numbers of binding sites thus identified were 2506, 22533, 55527, 17057, 8422, 6851, 3157, 15, 771, 1520, 3149, 28819, and 20975 for c-MYC, E2F1, ESRRB, KLF4, n-MYC, NANOG, OCT4, P300, SMAD1, STAT3, SUZ12, TCFCP2I1, and ZFX, respectively. For CTCF, the numbers of binding sites in genomic regions except for gene body were 16612.

### Repeat element analysis

Repeat element sequences were obtained from Repbase (http://www.girinst.org/repbase/). The number of the reads from ChIPseq data mapped on each repeat element was determined and corrected for the length of the repeat element and the number of reads for the input sample.

### Data access

Gene expression and sequencing data have been deposited in the DDBJ Sequence Read Archive (DRA) (http://trace.ddbj.nig.ac.jp/dra/index.html) under accession number DRP001103.

## Results

### Distribution of histone variants in genes

Because no ChIP antibody that reacts specifically with H3.1 or H3.2 is available, we prepared mouse ES cells (ZHBTc4) that expressed Flag-tagged H3.1, H3.2 and H3.3. We also prepared ES cells expressing Flag-tagged H2A variants to conduct ChIP with identical specificity and efficiency for H2A and H3 variants. Thus, we established seven cell lines that constitutively expressed Flag-tagged H2A, H2A.X, H2A.Z, mH2A, H3.1, H3.2 or H3.3.

**Figure 1:**
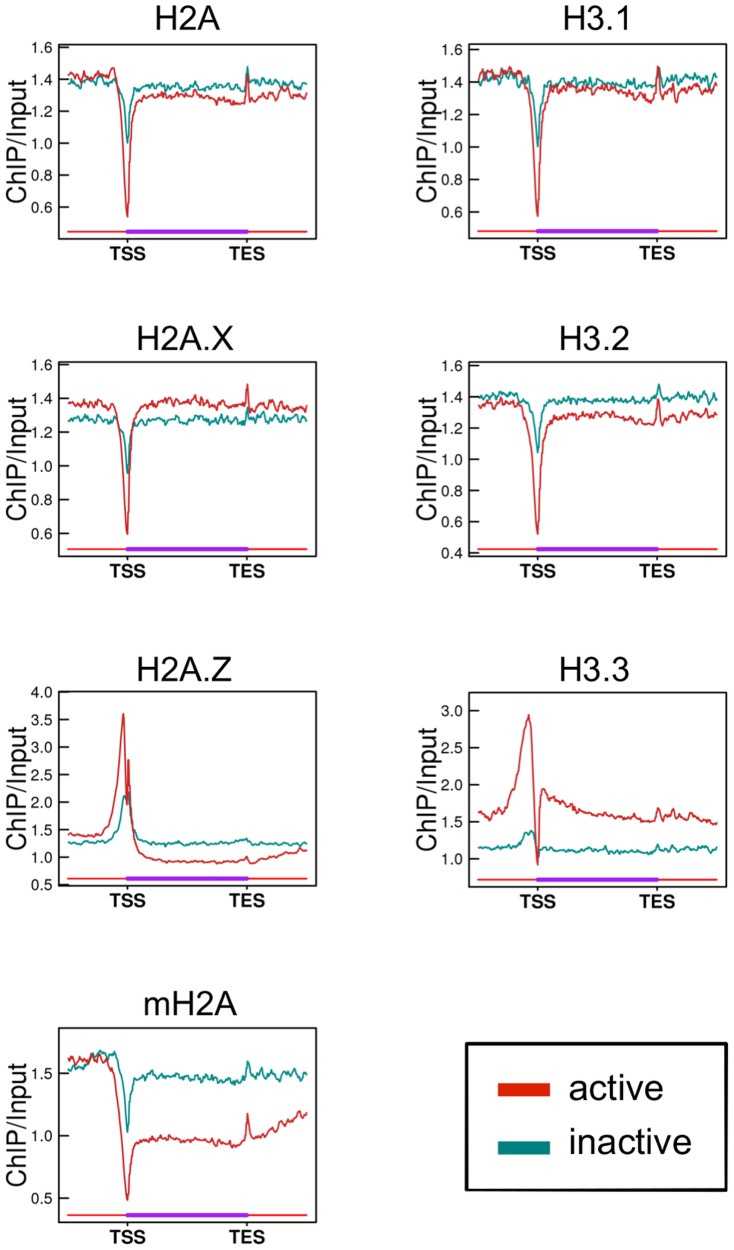
Enrichment of histone variants in active and inactive genes. Line graphs show the average enrichment (ChIP/input) of each histone variant in active (red) and inactive (dark cyan) genes. TSS and TES are the transcription start and end sites, respectively. The length of genes was normalized. In this analysis, genes of lengths <4 kb were excluded, as overlapping TSS and TES vicinities after length normalization would result in inaccuracies in the data of the gene bodies. In addition, genes whose distance from neighboring genes was <4 kb were also excluded to avoid overlapping of the flanking regions.

We first investigated the distribution of H2A and H3 variants both in genes and in their vicinities. Genes were classified as active or inactive based on RNA sequencing data (Figure S5). We found that all histone variants examined, except for H2A.Z, were markedly depleted at the TSS of active genes (Figure 1). In inactive genes, depletion at the TSS was also observed, except for H2A.Z and H3.3, but the degree of depletion was less than in active genes. In the H2A.Z and H3.3 ChIP data, peaks of abundant H2A.Z and H3.3 deposition were observed in the vicinity of the TSS. H3.3 was enriched in gene bodies as well as in the upstream regions of active genes compared to inactive genes. In contrast, mH2A enrichment was lower in the bodies of active genes than inactive genes, suggesting that chromatin in the bodies of active genes is depleted in mH2A. H2A.Z enrichment was also less in the bodies of active genes, while it was higher in the vicinity of the TSS.

To further assess the correlation between the expression level and the enrichment of histone variants in genes, genes were classified into six groups according to their expression levels (Figure S6). In these six groups, the enrichment of H3K4me3 and H3K27me3 at promoters, which are known to be positively and negatively correlated with gene expression level, respectively, and H3K36me3 enrichment in gene bodies, which is correlated with gene expression level [16], were altered in the expected manner (Figure S7, S8). In terms of histone variants, H3.3 and mH2A enrichment were positively and negatively correlated with gene expression, respectively, in both promoters and gene bodies (Figure S7, S8). A negative correlation was observed between gene expression level and H2A.Z enrichment in gene bodies (Figure S8). Interestingly, although H2A.Z enrichment at promoters was positively correlated with gene expression at a lower expression level (group 1–3), it was negatively correlated at a higher level (group 4–6) (Figure S7). These results suggest that H2A.Z, mH2A, and H3.3 deposition in chromatin plays an important role in the regulation of gene expression.

Chromatin structure and the regulation of transcription are known to be differ markedly between genes with promoters of high and low CpG densities [36]–[39]. To investigate the association of CpG density at promoters with the enrichment of histone variants in genes, we classified genes according to their promoter CpG density and expression level (Table S1). First, genes were classified into those with high, intermediate and low CpG density promoters (*i.e.*, HCP, ICP and LCP, respectively) according to Mikkelsen's criteria [34], and then as active and inactive using the criteria described above (Figure S5). Although in the gene body the enrichment of no variant differed markedly between genes with HCP and LCP promoters, enrichment of H2A.Z and H3.3 in the promoter regions differed markedly between these two types of genes (Figure S9, S10). The enrichment of H2A.Z was higher in genes with HCP promoters regardless of gene activity. In H3.3, enrichment was similar between active genes with LCP promoters and inactive genes with HCP promoters, and was higher in active genes with HCP promoters than the others. The enrichment of variants in the promoters was thus more dependent on the promoter type, HCP or LCP, than the gene expression level (Figure S9). The only exceptions were mH2A and H3.3 in the promoter region, enrichment of which seemed to be associated with gene expression (Figure S10).

**Figure 2:**
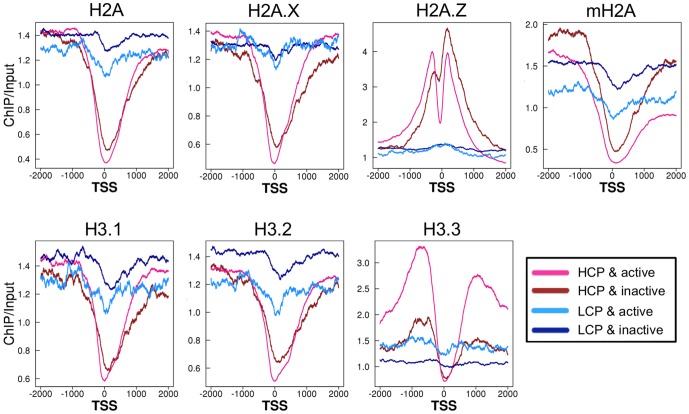
Enrichment of histone variants around the TSS with promoters of high and low CpG density. Line graphs show average enrichment of histone variants around the TSS of HCP & active, HCP & inactive, LCP & active and LCP & inactive genes.

A detailed analysis indicated that all H2A and H3 variants—with the exception of H2A.Z—were depleted from the nucleosome at the TSS of genes with HCP promoters, regardless of their expression levels (Figure 2). H2A.Z was also depleted from the nucleosome at the TSS in active, but not inactive, genes with HCP promoters. All of variants, except for H2A.Z, their levels were always higher in genes with LCP promoters than those with HCP ones, regardless of their gene expression levels. On both sides of the histone-depleted region at the TSS, H2A.Z was highly enriched in genes with HCP promoters, but not those with LCP promoters, regardless of their expression levels. Although H3.3 was also highly enriched in the vicinity of the TSS in genes with HCP promoters, it was present in only active genes. These results suggest that CpG density at the promoter, but not gene activity, is associated with the enrichment of the majority of H2A and H3 variants at the region around the TSS.

### Composition of histone variants in regulatory elements

**Figure 3:**
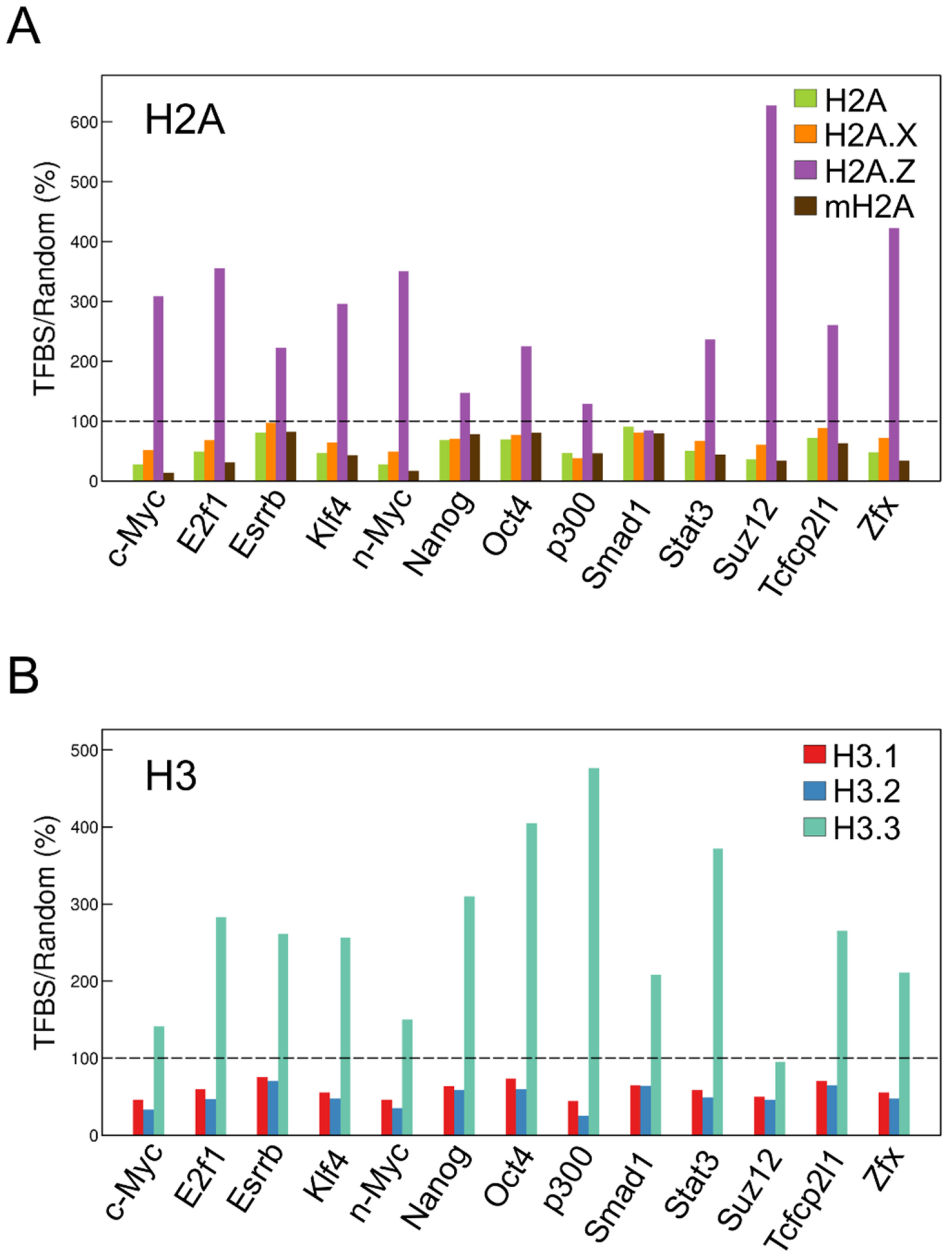
Enrichment of histone variants in enhancer regions. Bars represent the ratios (%) of enrichment of the histone H2A (A) and H3 (B) variants ±200 bp from the center of the transcription factor binding site (TFBS) as defined using Chen's TF ChIPseq data [35] to the average enrichment in 5,000 400-bp regions randomly selected from the whole genome, excluding TFBSs. The dotted line indicates 100%, representing the average value of the enrichment in randomly selected regions.

Many regulatory elements have specific functions in the genome. We thus investigated the enrichment of H2A and H3 variants in these elements to evaluate the involvement of histone variants in their functions. Both H2A.Z and H3.3 were enriched in all of enhancers defined as transcription factor binding sites (TFBSs) [40], except for *Smad1*, suggesting that the nucleosomes at enhancer elements comprise mainly H2A.Z and H3.3 (Figure 3).

**Figure 4:**
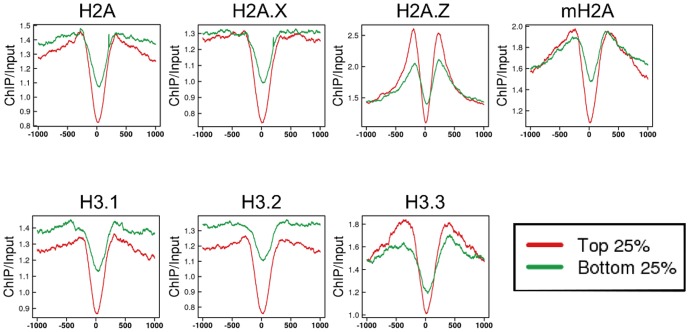
Enrichment of histone variants in insulator regions. Profile of the average enrichment of H2A (upper) and H3 (lower) variants in insulator regions defined as CTCF binding sites in genomic regions except for gene body obtained from Chen's ChIPseq data [35]. The 0 position is the center of the CTCF binding site. CTCF binding sites were ordered based on the amount of CTCF bound; the average enrichment of histone variants in the top (red) and bottom (green) 25% is shown.

Next, we examined histone variant enrichment in insulators. Insulators bind to CCCTC-binding factor (CTCF), which contributes to higher-order chromatin architecture for long-range interactions [41], [42]. These interactions regulate enhancer-promoter binding, and therefore are involved in the regulation of gene expression [43]–[45]. CTCF also contributes to pol II stalling and splicing in bodies of genes [46]. Therefore, we defined insulators as the sites to which CTCF was bound in genomic regions except for gene body (*see* Materials and Methods) and compared the enrichment of histone variants at sites with the highest and lowest quartile of bound CTCF. All variants were absent from the CTCF binding sites in both the highest and lowest quartiles, although the extent of the removal was greater in the highest quartile (Figure 4), suggesting that nucleosomes are almost completely depleted from insulator elements when they bind CTCF. In addition, on both sides of the binding site, abundant H2A.Z deposition, as evidenced by the peak height, was observed. The peaks for H3.3 and mH2A were slightly wider at these regions. The enrichment of H3.1 and H3.2 was low in a wide region around the CTCF binding sites.

**Figure 5:**
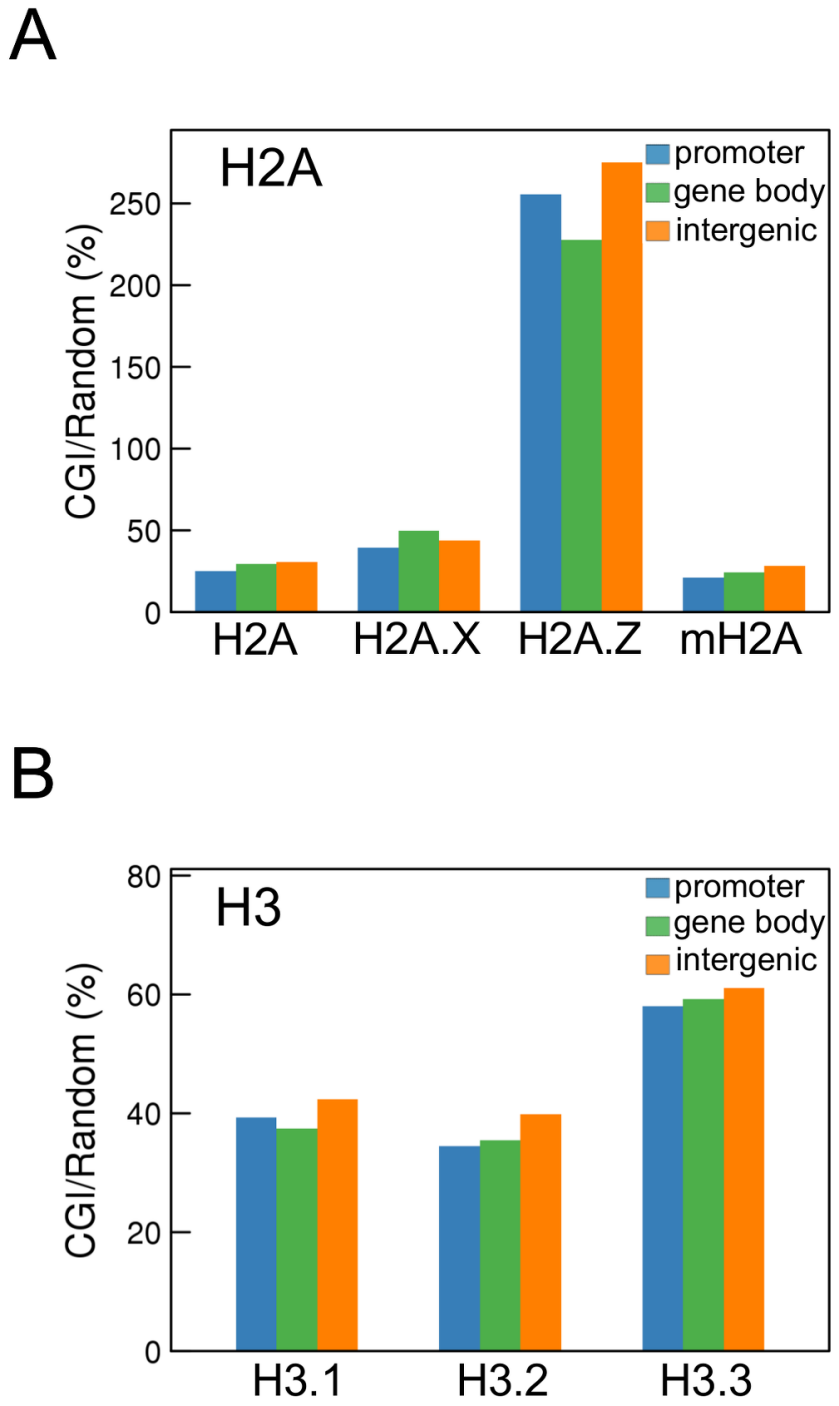
Enrichment of histone variants in CpG islands (CGIs). The enrichment of histone H2A (A) and H3 (B) variants in CGIs defined in the UCSC Genome Bioinformatics Site was determined in promoters (blue), gene bodies (green), and intergenic regions (orange). The ratios (%) of the enrichment in CGIs to the averaged enrichment in 5,000 randomly selected 656-bp regions, which is the average length of all CGIs analyzed, from the whole genome excluding CGIs were calculated.

Many genes have CpG islands (CGIs) in their upstream regulatory regions; moreover, a number of CGIs are present in gene bodies and intergenic regions [47]. We found that H2A.Z was more enriched in CGIs compared with other H2A variants (Figure 5A). Among the H3 variants, H3.3 was relatively highly enriched in CGIs (Figure 5B). This enrichment of H2A.Z and H3.3 at CGIs was true for all genome regions (*i.e.*, promoters, gene bodies, and intergenic regions), suggesting that their enrichment at CGIs is not regulated by a particular epigenetic environment but is dependent only on the presence of a CG-rich DNA sequence.

### Composition of histone variants in repeat elements

**Figure 6:**
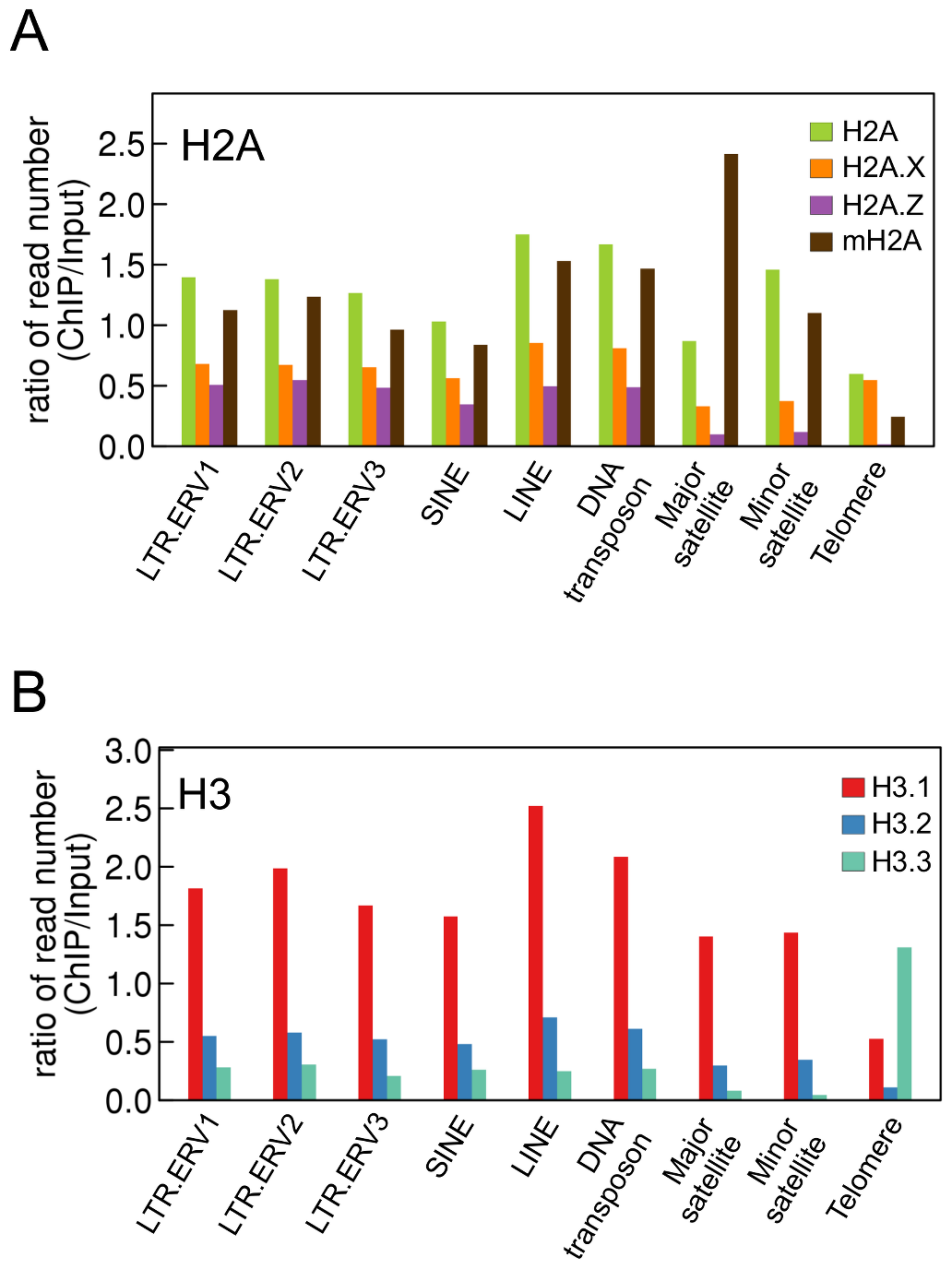
Enrichment of histone variants in repeat elements. Bars represent the ratios of the number of ChIP reads to that of input reads in repeat elements.

Repeat elements are generally enriched in H3K9me3 and H4K20me3, and are constitutively heterochromatic [34]. We found that of the H2A and H3 variants, H2A, mH2A, and H3.1 were relatively enriched in most repeat elements (Figure 6). The only exception was the telomeres, in which H3.3 was enriched, but where no particular H2A variant was enriched. Remarkably, H2A.Z enrichment in this region was extremely low.

### Preferential combination of H2A and H3 variants in various genomic regions

**Figure 7:**
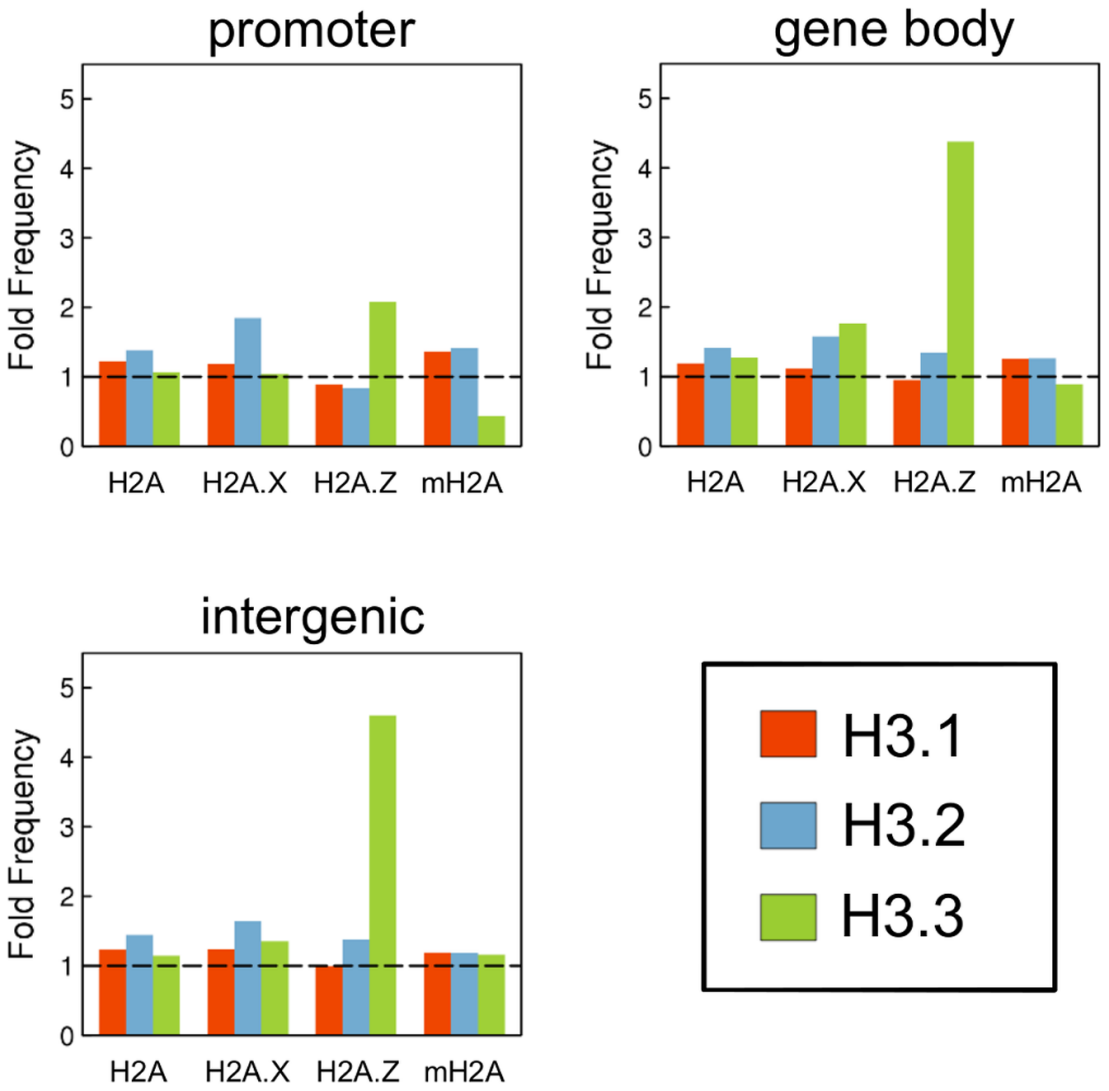
Preferred combination of H2A and H3 variants in various genome regions. The genome was divided into 150 nucleotide bins, and the bins in which a particular variant was significantly enriched were determined as described in the Materials and Methods (*see* Figure S4). The frequency of bins in which two H2A and H3 variants were simultaneously enriched was determined for all combinations of H2A and H3 variants. Bars represent the ratios of the frequencies determined as described above to those of the expected values calculated from the number of bins in which each variant was enriched. Therefore, values >1.0 indicate a preference for that particular H2A and H3 variant combination.

To investigate the existence of a preferential combination of H2A and H3 variants comprising nucleosomes in various genome regions, we compartmentalized the genome into 150-bp bins, and identified those in which the particular histone H2A or H3 variant was enriched (Figure S4). Then, we determined the frequencies of bins in which a pair of H2A and H3 variants was enriched simultaneously. As shown in Figure 7, the frequency of bins in which H2A.Z and H3.3 enrichment overlapped was high in all genome regions. These results suggest that nucleosomes tend to comprise a combination of H2A.Z and H3.3 variants, and that this property is independent of the genome region.

**Figure 8:**
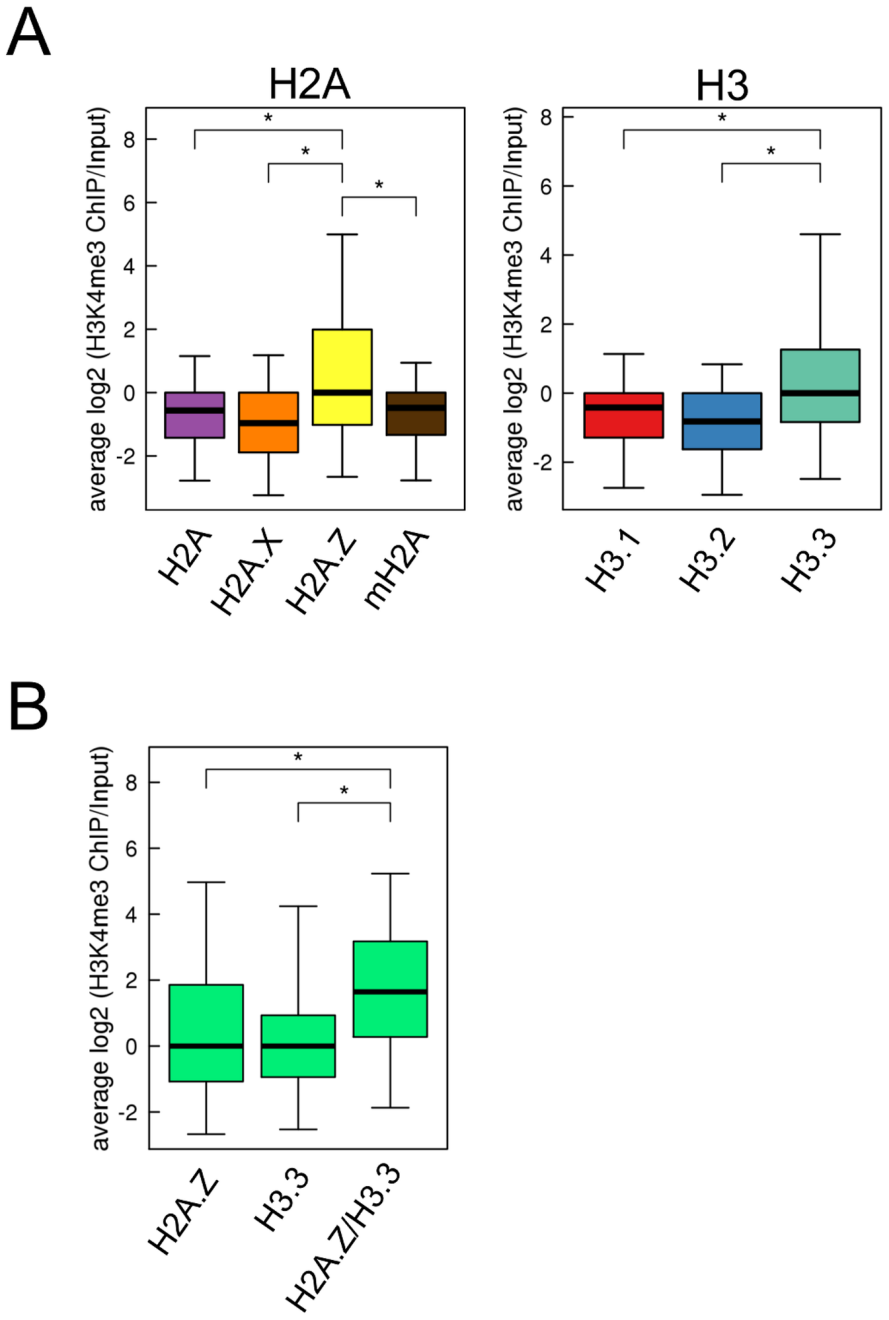
H3K4me3 level in H2A.Z- and H3.3-enriched regions. The genome was divided into 150 nucleotide bins, and the bins in which a particular variant was significantly enriched was determined as described in Materials and Methods (*see* Figure S4). (A) Boxplot of H3K4me3 levels in H2A-and-H3-variant-enriched bins. (B) Boxplot of H3K4me3 levels in H2A.Z- or H3.3-, and both H2A.Z and H3.3 (H2A.Z/H3.3)-enriched bins. The bottom and top of the box are the 25th and 75th percentile, respectively, and the upper and lower whiskers represent 2.5th and 97.5th percentile, respectively. *P*-values were calculated using Mann–Whitney U-tests; (*) *P*<0.001.

Both H2A.Z and H3.3 are associated with H3K4me3 [7], [48]; thus, we investigated whether the combination of H2A.Z and H3.3 influences the level of H3K4me3. Consistent with previous reports, the H3K4me3 level was highest in the H2A.Z- and H3.3-enriched bins (Figure 8A). The H3K4me3 level was higher in bins in which both H2A.Z and H3.3 were enriched simultaneously than those in which only H2A.Z or H3.3 was enriched (Figure 8B). These results suggest that nucleosomes composed of H2A.Z and H3.3 are preferentially subjected to the H3K4me3 modification.

## Discussion

Several reports of the genome-wide distribution of various histone variants have been published [3], [4], [7], [22], [26], [28], [31], [49]. However, most examined a single histone variant. In the present study, we investigated the majority of H2A and H3 variants expressed in mouse ES cells by genome-wide analysis using ChIP sequencing, which revealed enrichment of H2A and H3 variants in various genome regions (Figure 1–6). Furthermore, analysis of the combinations of H2A and H3 variants suggests that H2A.Z and H3.3 preferentially associate with each other to generate nucleosomes throughout the genome (Figure 7). Such data could only be obtained by simultaneous analysis of several variants. During this study, a report of the genome-wide distribution of most H2A variants (*i.e.*, H2A, H2A.Z, mH2A, and H2A.Bbd) was published [50]; however, H2A.X was not included. Of all H2A variants, H2A.X is evolutionarily closest to H2A. H2A.X and H2A are not distinguished as separate proteins in phylogenetically ancient species (*e.g.*, insects and fungi). Therefore, H2A and H2A.X must be investigated simultaneously to understand their roles in vertebrate genomic function. In addition, the amino acid sequence of H3.1 is similar to that of H3.2, and is present only in mammals. Because no antibody that can distinguish these proteins is available, the difference in their genome distribution and function has not been determined. Here we report that the genome distributions of H2A and H2A.X, and H3.1 and H3.2 differ, suggesting that each of these variants has a specific function that is different from their counterparts.

We found that H3.1 but not H3.2 was enriched in repeat elements other than telomeres (Figure 6B). These elements are enriched in H3K9me3 [34], [51], and so constitutive heterochromatin is generated [52], [53]. In addition, H3K9me2 is enriched at centromere minor repeats [54], [55]. H3.1 is preferentially modified with H3K9me2 and H3K9me3 as compared to other H3 variants [24], [25]. Therefore, repeat elements are enriched in H3.1 with H3K9me2 and H3K9me3, which contributes to heterochromatin formation. Furthermore, enrichment of H2A and H2A.X was different in repeat elements other than telomeres (Figure 6A). These data demonstrate the difference between H2A and H2A.X as well as H3.1 and H3.2, and suggest that nucleosomes composed of H2A and H3.1 contribute to the formation of constitutive heterochromatin at repeat elements.

Histone variants play important roles in the regulation of transcription. Previous reports showed that H2A.Z and H3.3 enrichment in promoters and gene bodies was well correlated with the expression levels of the genes [3], [7], [16], and that mH2A enrichment in gene bodies was inversely correlated with gene expression [6]. In the present study, the enrichment of these variants in promoters and gene bodies was correlated with gene expression levels, as reported previously (Figure 1 and Figure S7, S8). H3.2 enrichment is negatively correlated with gene expression in *Arabidopsis thaliana* [28]. However, this finding was not replicated here (Figure S7, S8). This is consistent with the study by Goldberg *et al*. (2010), who reported that H3.2 enrichment was not correlated with gene expression in mouse ES cells. Therefore, the role of H3.2 in gene expression seems to differ between mice and plants.

Previous studies have shown that nucleosomes are depleted at the TSSs of actively expressed genes [56]–[58]. However, one recent report showed that TSSs are sensitive to MNase and that the sensitivity is more dependent on CpG density in promoters than on gene expression [59], [60], which suggests that nucleosomes are unstable or depleted at TSSs of HCP genes. Supporting this hypothesis is our finding that almost all histone variants were depleted at TSSs of HCP, but not LCP, genes regardless of their expression levels (Figure 2). The discrepancy between previous, and recent studies—including ours—may be due to the difference in the occupancies of genes with HCP and LCP promoters between the active and inactive gene groups. Genes with HCP and LCP promoters comprise major proportions of active and inactive genes, respectively (Table S1). Therefore, the results of previous analyses of active and inactive genes likely reflect genes with HCP and LCP promoters, which represent active and inactive genes, respectively.

Nucleosomes containing H2A.Z and H3.3 are preferentially subjected to the H3K4me3 modification. Previous studies showed that regions of H2A.Z enrichment overlapped substantially with those of H3K4me3 in promoter and enhancer regions [48]. The distribution of H3.3-enriched regions is similar to that of H3K4me3 [7]. Consistent with these results, we showed that H3K4me3 enrichment in nucleosomes containing H2A.Z and H3.3 was higher than in those containing other H2A and H3 variants (Figure 8A). Furthermore, we suggest that the nucleosomes composed of H2A.Z and H3.3 were more highly enriched in H3K4me3 than those containing only H2A.Z or H3.3 (Figure 8B). Thus, the combination of H3.3 and H2A.Z is important in terms of constituting the structure of nucleosomes that are preferentially subjected to H3K4me3, and are involved in the formation of transcriptionally active chromatin. Therefore, information from only one of each H2A and H3 variant would not be enough to understand the structure of nucleosomes and chromatin, and analyzing the combination of H2A and H3 variants composing the nucleosomes is important.

H2A.Z and H3.3 were enriched in regulatory elements (*i.e.*, enhancers, insulators, and CGIs; Figure 3, 4, 5). Various chromatin proteins bind to these regions, including the transcription factors, CTCF and Cfp1, which recruit histone modification enzymes [61], [62]. Therefore, H2A.Z and H3.3 might be recruited to these regions to form nucleosomes that are readily accessible to chromatin proteins. In insulator regions, mH2A peaks were slightly wider. Insulators form chromatin loop, and contribute to higher-order chromatin [41], [42]. Therefore, mH2A, which contributes to formation of chromatin loop, may be abundant both up- and downstream of insulator regions.

**Figure 9:**
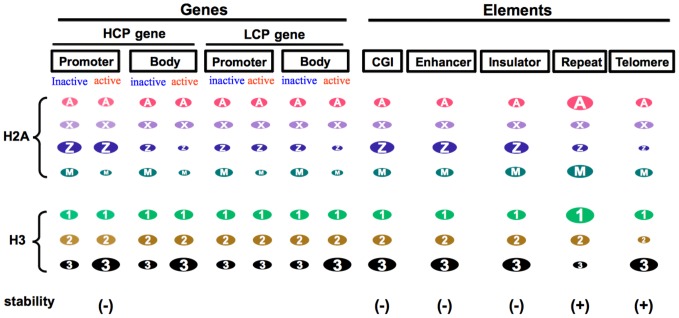
Schematic view of histone variant enrichment in various genome regions. Relative enrichment of each histone variant in various genome regions is indicated by the ellipse magnitude. Stability (+) and (−) indicate stable and unstable chromatin, respectively. Chromatin was unstable only in regions enriched for both H2A.Z and H3.3.

Previous studies suggested that nucleosomes containing both H2A.Z and H3.3 compose unstable chromatin. Jin and Felsenfeld [32] reported that nucleosomes containing both of these variants were more highly unstable than those containing other combinations of the H2A and H3 variants under high-salt conditions *in vitro*. Genome-wide analysis of the distribution of H2A.Z and H3.3 revealed that both of these variants are enriched around the TSSs of active genes, enhancers and insulators, which suggests that in these regions, nucleosomes are so unstable that the variants are easily removed and displaced by DNA-binding proteins (*e.g.*, transcription factors and CTCF) [26]. In the present study, we found that both H2A.Z and H3.3 are abundant in genome regions known to have unstable chromatin (*i.e.*, the promoters of active HCP genes, insulators, enhancers and CGIs; Figure 9). Ramirez-Carrozzi [63] reported that the expression of LCP genes depended on the SWI/SNF chromatin remodeling factors, whereas that of HCP genes was independent of SWI/SNF, suggesting that the chromatin of HCP genes is so unstable that transcription factors can easily access DNA without remodeling the chromatin structure. It has also been shown that the turnover rate of H3.3 was high around the TSS of active genes [64]. Insulators, enhancers and CGIs are DNase I hypersensitive, which suggests that the chromatin of these elements is loosened and unstable [65]–[68]. In contrast, it is interesting that in regions in which only H3.3 is enriched, chromatin is not unstable but rather stable (Figure 9). In the bodies of transcribed genes in which only H3.3 is enriched, chromatin is so stable that histone exchange is suppressed, which prevents the cryptic initiation of transcription of the gene bodies [69]–[72], although a report showed that the turnover of chromatin proteins was rapid in the bodies of active genes [73]. In telomeres, in which stable constitutive heterochromatin is formed [74], only H3.3 is enriched. Therefore, the histone variant composition is an important determinant of chromatin stability, which is associated with various genomic functions.

In summary, we clarified the histone variant composition of various genome regions by genome-wide analysis of the majority of H2A and H3 variants expressed in mouse ES cells. We found that in HCP, but not LCP, genes, regardless of their expression levels, most of the variants were depleted at the TSS but that H2A.Z and H3.3 were enriched in the vicinity of the TSS. H2A.Z and H3.3 were also enriched in enhancers, CpG islands and insulators. We found that H2A and H3.1 were enriched in repeat elements, which is the first knowledge of the differential distribution between H2A and H2A.X, and between H3.1 and H3.2 on specific genome regions. The analysis of the combinations of H2A and H3 variants that comprise nucleosomes revealed that the frequency of the H2A.Z and H3.3 combination was high throughout the genome when compared to other combinations, and that nucleosomes containing this combination were preferentially trimethylated at the lysine 4 residue. None of these results could have been obtained without simultaneous analysis of the majority of histone variants. Taken together, these data suggest that specific combinations of histone variants influence the regulation of various genomic functions (*e.g.*, gene expression and heterochromatin formation) and histone modifications.

## Supporting Information

Figure S1 **The genome distribution pattern of Flag-H2A.X among various cell lines.** The enrichment of Flag-H2A.X in four genome regions (*i.e.*, *Nanog* promoter, *Cdx* 3′UTR, *Prmt1* promoter and *Sry* promoter) was examined in four cell lines expressing Flag-H2A.X by ChIP-qPCR.
(TIF)

Figure S2 **Morphology of ES cells expressing Flag-tagged histone variants.** ZHBTc4 cells expressing Flag-tagged H2A, H2A.X, H2A.Z, macroH2A (mH2A), H3.1, H3.2 or H3.3, and wild-type (WT) cells, were observed before (0 h) and after (72 h) induction of differentiation.
(TIF)

Figure S3 **Clustering analysis of gene expression profiles in ES cells expressing Flag-histone variants and wild-type cells before and after the induction of differentiation.** cDNA microarray data sets of wild-type ZHBTc4 cells before (WT_0 h) and 24, 48, 72, and 96 h after induction of differentiation (WT_24 h, WT_48 h, WT_72 h, and WT_96 h, respectively) and cells expressing H2A, H2A.X, H2A.Z, macroH2A (mH2A), H3.1, H3.2 or H3.3 without differentiation induction were subjected to hierarchical clustering. Results are shown as a tree view.
(TIF)

Figure S4 **Identification of the regions in which a particular histone H2A or H3 variant is enriched.** As an example, the identification of the bin in which H2A.Z is enriched is shown.
(TIF)

Figure S5 **Histogram of the expression levels of protein-coding genes in mouse ES cells.** The expression levels of coding genes were obtained from the RNA sequence data. The histogram of gene expression levels was smoothed by kernel density estimation, which is depicted by bimodal peaks. The genes in the first and second peaks are defined as inactive and active, respectively.
(TIF)

Figure S6 **Classification of genes according to expression level.** The expression level of coding genes was obtained from the RNA sequence data. The histogram of gene expression levels was smoothed by kernel density estimation. The genes were classified into six groups according to their expression levels. Groups 1, 2, 3, 4, 5 and 6 contained 6728, 447, 1161, 2526, 2760 and 1009 genes, respectively.
(TIF)

Figure S7 **Correlation between the expression level and enrichment of histone variants in gene promoters.** Genes were classified into six groups according to their expression levels as shown in Figure S6, and the enrichment of histone variants and histone modifications in the promoters (TSS±2 kb) of genes in each group are shown as box plots. The bottom and top of the box are the 25th and 75th percentile, respectively, and the upper and lower whiskers represent 2.5th and 97.5th percentile, respectively.
(TIF)

Figure S8 **Correlation between the expression level and enrichment of histone variants in gene bodies.** Genes were classified into six groups according to their expression levels as shown in Figure S6, and the enrichment of histone variants and histone modifications in the gene body (TSS+2 kb∼TES) in each group are shown as box plots.
(TIF)

Figure S9 **Enrichment of histone variants in the promoters of HCP and LCP genes.** Genes were classified into HCP & active, HCP & inactive, LCP & active, and LCP & inactive on the basis of CpG density at the promoter and gene expression levels. Enrichment of histone variants at promoters (TSS±2 kb) in each group is shown as a box plot.
(TIF)

Figure S10 **Enrichment of histone variants in the bodies of HCP and LCP genes.** Genes were classified into HCP & active, HCP & inactive, LCP & active, and LCP & inactive on the basis of CpG density at the promoter and gene expression levels. Enrichment of histone variants at gene bodies (TSS+2 kb∼TES) in each group is shown as a box plot.
(TIF)

Table S1 **Classification of the genes by their expression levels and the CpG density at promoter.**
(TIF)

Table S2 **The number of reads uniquely mapped to the genome for all ChIPseq and Inputseq data sets.**
(TIF)
