# Supplementary figures and images for: Genome-Wide Analysis of the Chromatin Composition of Histone H2A and H3 Variants in Mouse Embryonic Stem Cells
Source: PLoS One. 2014 Mar 21;9(3):e92689. doi: 10.1371/journal.pone.0092689 (PMC3962432; doi:10.1371/journal.pone.0092689)

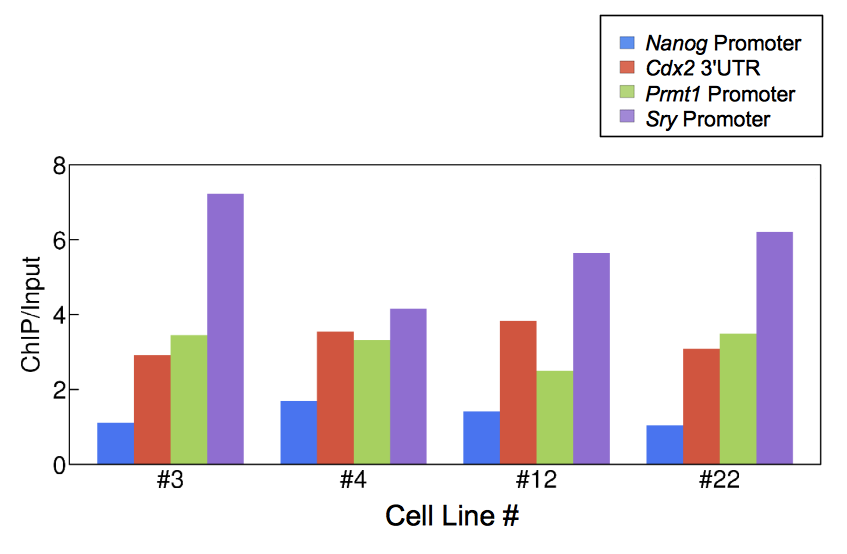

Supplement: Figure S1 — The genome distribution pattern of Flag-H2A.X among various cell lines. The enrichment of Flag-H2A.X in four genome regions (i.e., Nanog promoter, Cdx 3′UTR, Prmt1 promoter and Sry promoter) was examined in four cell lines expressing Flag-H2A.X by ChIP-qPCR. (TIF) [file pone.0092689.s001.tif]

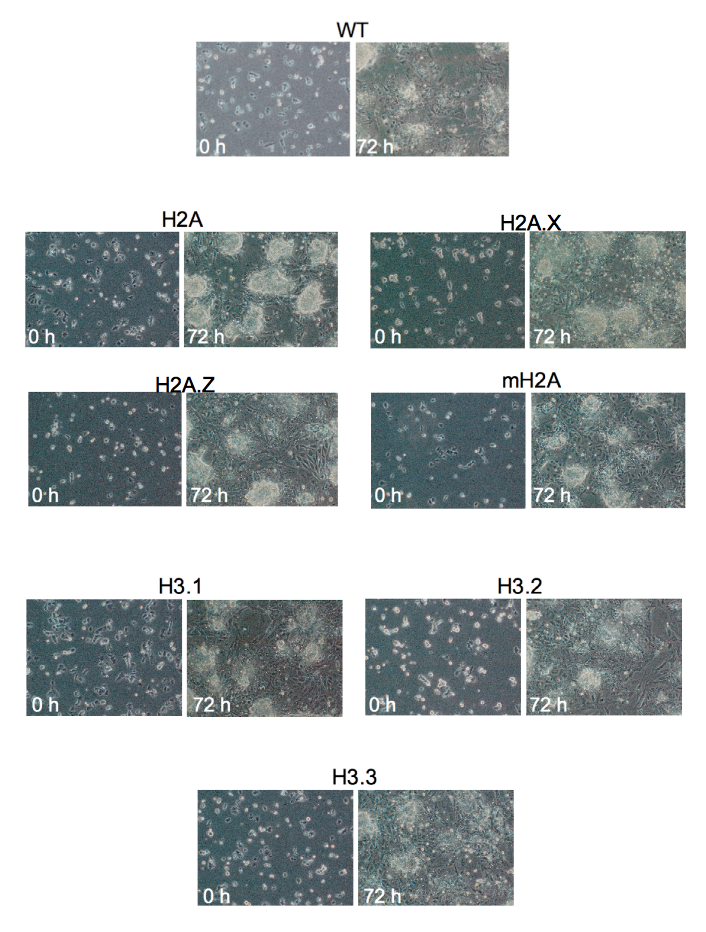

Supplement: Figure S2 — Morphology of ES cells expressing Flag-tagged histone variants. ZHBTc4 cells expressing Flag-tagged H2A, H2A.X, H2A.Z, macroH2A (mH2A), H3.1, H3.2 or H3.3, and wild-type (WT) cells, were observed before (0 h) and after (72 h) induction of differentiation. (TIF) [file pone.0092689.s002.tif]

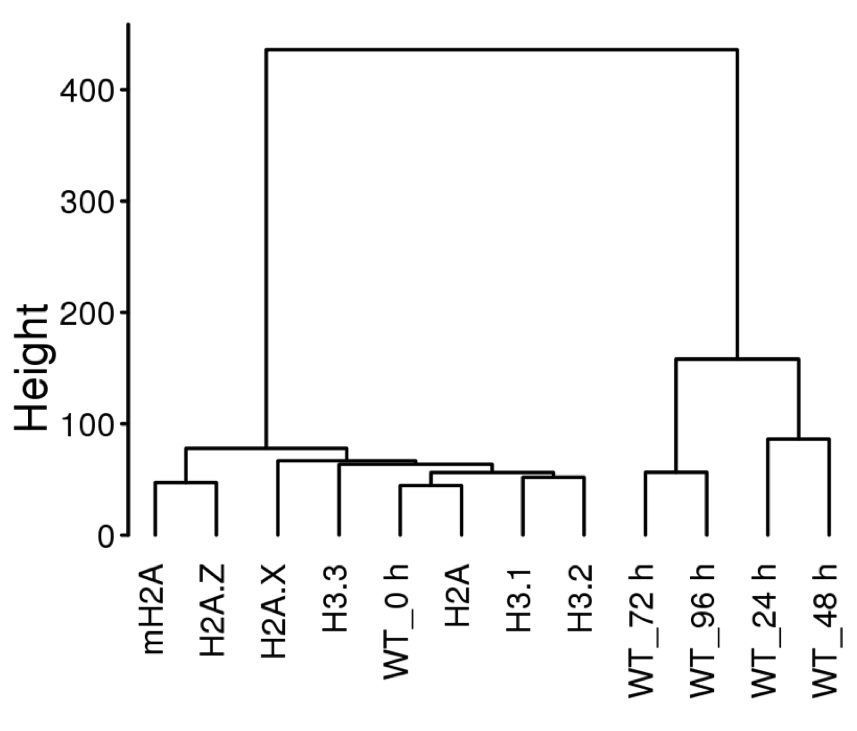

Supplement: Figure S3 — Clustering analysis of gene expression profiles in ES cells expressing Flag-histone variants and wild-type cells before and after the induction of differentiation. cDNA microarray data sets of wild-type ZHBTc4 cells before (WT_0 h) and 24, 48, 72, and 96 h after induction of differentiation (WT_24 h, WT_48 h, WT_72 h, and WT_96 h, respectively) and cells expressing H2A, H2A.X, H2A.Z, macroH2A (mH2A), H3.1, H3.2 or H3.3 without differentiation induction were subjected to hierarchical clustering. Results are shown as a tree view. (TIF) [file pone.0092689.s003.tif]

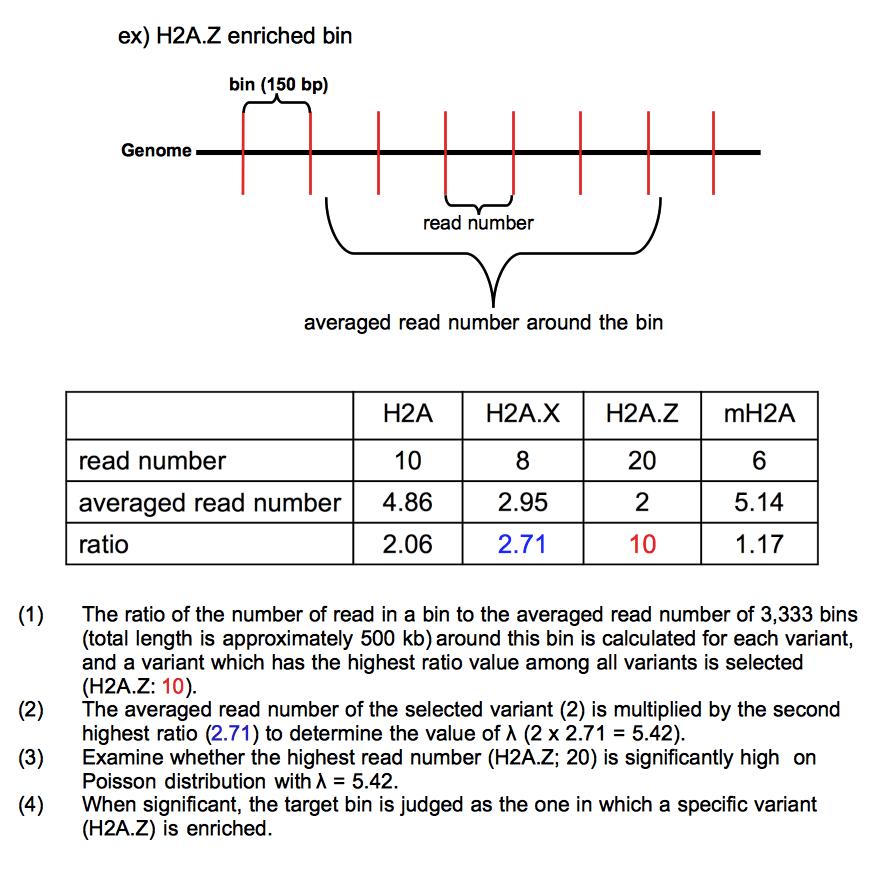

Supplement: Figure S4 — Identification of the regions in which a particular histone H2A or H3 variant is enriched. As an example, the identification of the bin in which H2A.Z is enriched is shown. (TIF) [file pone.0092689.s004.tif]

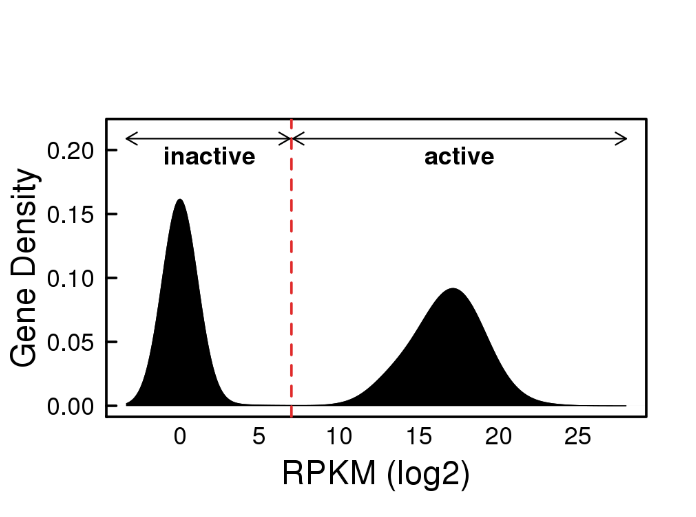

Supplement: Figure S5 — Histogram of the expression levels of protein-coding genes in mouse ES cells. The expression levels of coding genes were obtained from the RNA sequence data. The histogram of gene expression levels was smoothed by kernel density estimation, which is depicted by bimodal peaks. The genes in the first and second peaks are defined as inactive and active, respectively. (TIF) [file pone.0092689.s005.tif]

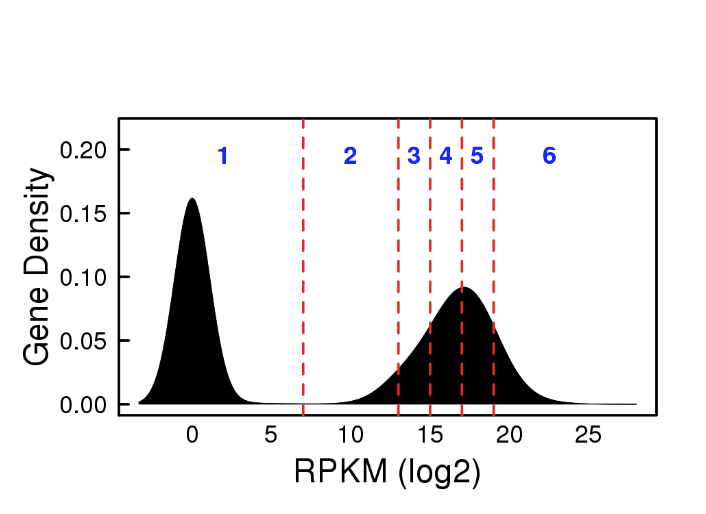

Supplement: Figure S6 — Classification of genes according to expression level. The expression level of coding genes was obtained from the RNA sequence data. The histogram of gene expression levels was smoothed by kernel density estimation. The genes were classified into six groups according to their expression levels. Groups 1, 2, 3, 4, 5 and 6 contained 6728, 447, 1161, 2526, 2760 and 1009 genes, respectively. (TIF) [file pone.0092689.s006.tif]

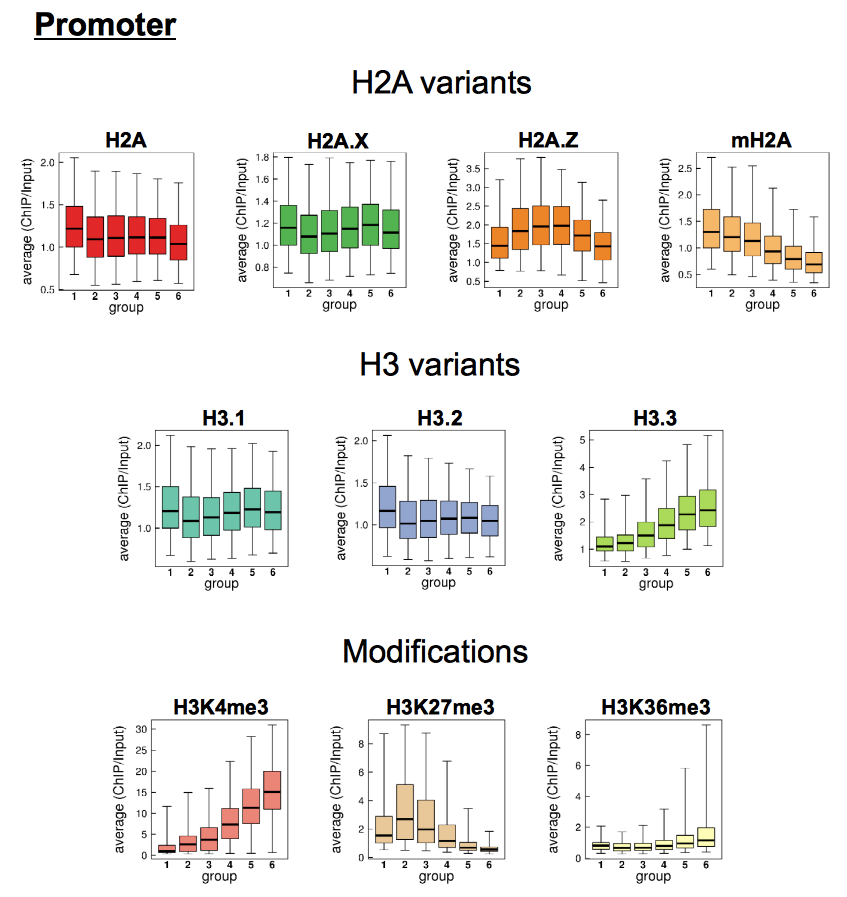

Supplement: Figure S7 — Correlation between the expression level and enrichment of histone variants in gene promoters. Genes were classified into six groups according to their expression levels as shown in Figure S6, and the enrichment of histone variants and histone modifications in the promoters (TSS±2 kb) of genes in each group are shown as box plots. The bottom and top of the box are the 25th and 75th percentile, respectively, and the upper and lower whiskers represent 2.5th and 97.5th percentile, respectively. (TIF) [file pone.0092689.s007.tif]

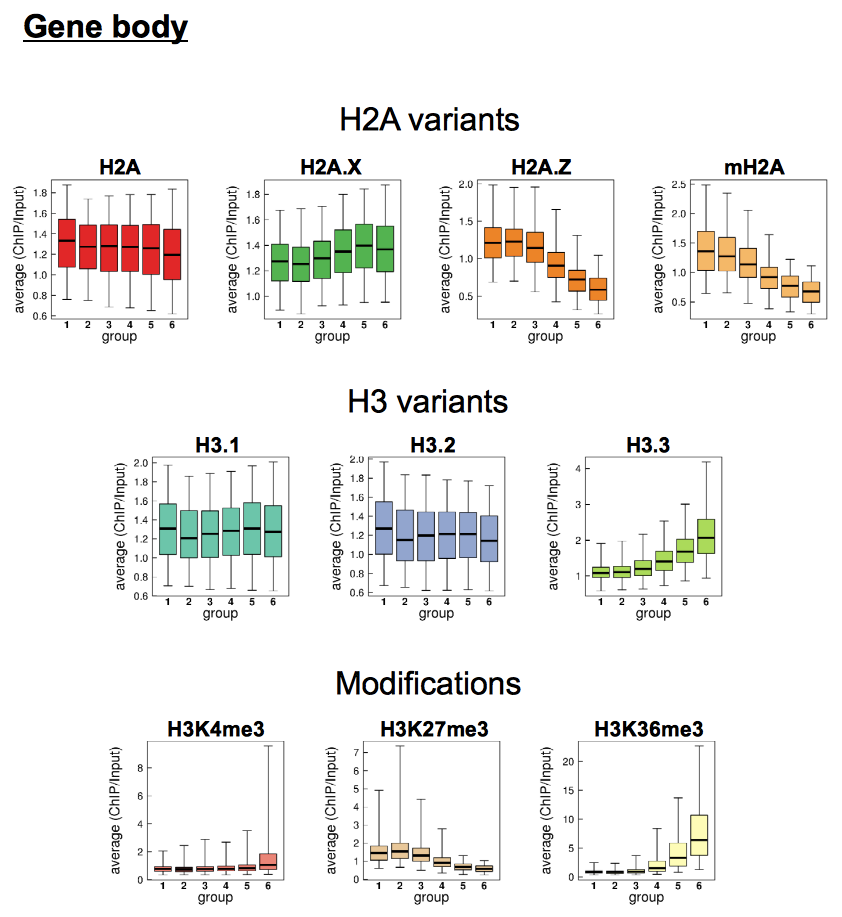

Supplement: Figure S8 — Correlation between the expression level and enrichment of histone variants in gene bodies. Genes were classified into six groups according to their expression levels as shown in Figure S6, and the enrichment of histone variants and histone modifications in the gene body (TSS+2 kb∼TES) in each group are shown as box plots. (TIF) [file pone.0092689.s008.tif]

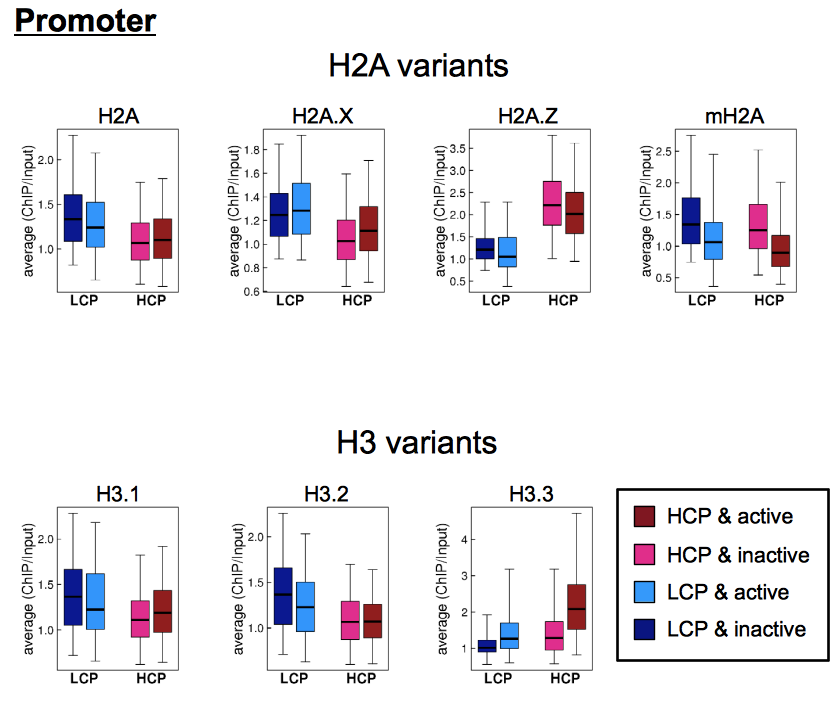

Supplement: Figure S9 — Enrichment of histone variants in the promoters of HCP and LCP genes. Genes were classified into HCP & active, HCP & inactive, LCP & active, and LCP & inactive on the basis of CpG density at the promoter and gene expression levels. Enrichment of histone variants at promoters (TSS±2 kb) in each group is shown as a box plot. (TIF) [file pone.0092689.s009.tif]

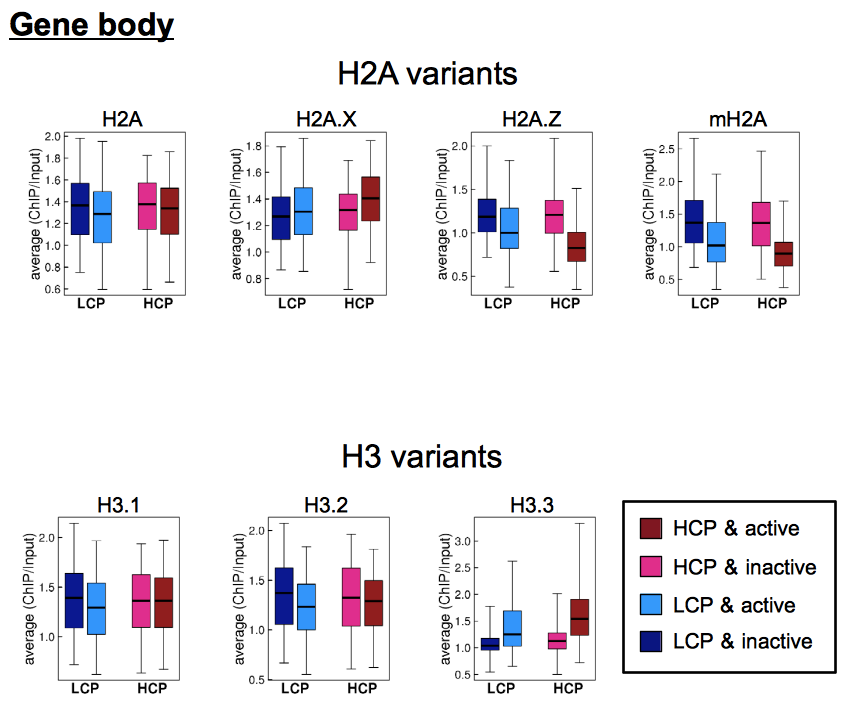

Supplement: Figure S10 — Enrichment of histone variants in the bodies of HCP and LCP genes. Genes were classified into HCP & active, HCP & inactive, LCP & active, and LCP & inactive on the basis of CpG density at the promoter and gene expression levels. Enrichment of histone variants at gene bodies (TSS+2 kb∼TES) in each group is shown as a box plot. (TIF) [file pone.0092689.s010.tif]

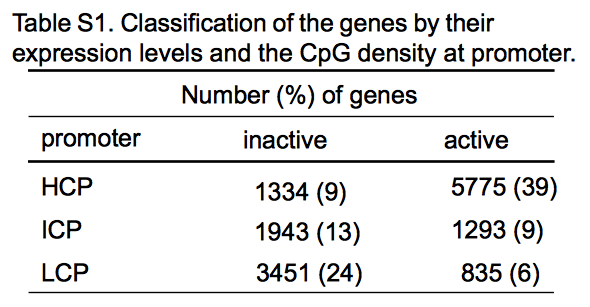

Supplement: Table S1 — Classification of the genes by their expression levels and the CpG density at promoter. (TIF) [file pone.0092689.s011.tif]

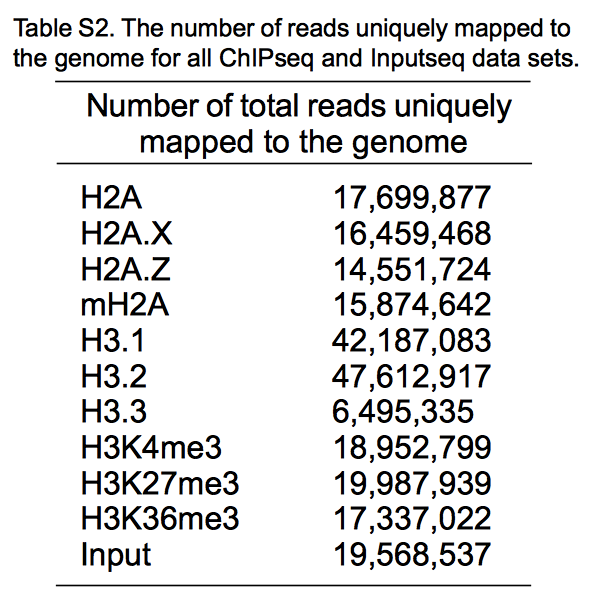

Supplement: Table S2 — The number of reads uniquely mapped to the genome for all ChIPseq and Inputseq data sets. (TIF) [file pone.0092689.s012.tif]
